# The DizzyQuest: to have or not to have… a vertigo attack?

**DOI:** 10.1007/s00415-020-10043-x

**Published:** 2020-07-11

**Authors:** L. E. G. H. de Joode, E. C. Martin, J. J. A. Stultiens, C. Leue, P. Delespaul, F. Peeters, A. Erdkamp, S. van de Weijer, H. Blom, T. Bruintjes, A. Zwergal, E. Grill, N. Guinand, A. Perez-Fornos, M. R. van de Berg, J. Widdershoven, H. Kingma, R. van de Berg

**Affiliations:** 1 Division of Balance Disorders, Department of Otorhinolaryngology and Head and Neck Surgery, Maastricht University Medical Center, Maastricht, Netherlands; 2 Department of Psychiatry and Neuropsychology, School for Mental Health and Neuroscience, Maastricht University Medical Center, Maastricht, Netherlands; 3 Department of Clinical Psychological Science, Faculty of Psychology and Neuroscience, Maastricht University, Maastricht, Netherlands; 4 mHealth, Maastricht University Medical Center, Maastricht, Netherlands; 5 Department of ENT, HagaZiekenhuis, The Hague, Netherlands; 6 Apeldoorns duizeligheidscentrum, Gelre ziekenhuizen, Apeldoorn, Netherlands; 7 Department of Neurology, Ludwig-Maximilians-University of Munich, Munich, Germany; 8 Department of Medical Informatics, Ludwig-Maximilians-University of Munich, Munich, Germany; 9 Service of Otorhinolaryngology Head and Neck Surgery, Department of Clinical Neurosciences, Geneva University Hospitals, Geneva, Switzerland; 10 Faculty of Physics, Tomsk State Research University, Tomsk, Russia

**Keywords:** DizzyQuest, Experience sampling, Vestibular disorders

## Abstract

**Background:**

The DizzyQuest, an app-based vestibular diary, provides the opportunity to capture the number and nature of vertigo attacks in daily life. To accomplish this, the DizzyQuest provides different strategies: event sampling using an attack questionnaire, and time sampling using an evening questionnaire. Objective of this study was to investigate whether the number and nature of reported vertigo attacks was comparable between the two questionnaires.

**Methods:**

Fifty-seven patients, who reported vertigo attacks, used the DizzyQuest for on average 24 days. The number and nature (including symptoms, triggers and duration) of vertigo attacks were compared between the attack and the evening questionnaire.

**Results:**

The attack questionnaire was used 192 times. In contrast, at least 749 new vertigo attacks were reported in 446 evening questionnaires. A vertigo attack was not always reported in both questionnaires during the same day. Vertigo attacks that were most likely captured by both questionnaires were not always reported the same in both questionnaires regarding triggers and duration.

**Conclusion:**

Event sampling using an attack questionnaire has low recall bias and, therefore, reliably captures the nature of the attack, but induces a risk of under-sampling. Time sampling using an evening questionnaire suffers from recall bias, but seems more likely to capture less discrete vertigo attacks and it facilitates registration of the absence of vertigo attacks. Depending on the clinical or research question, the right strategy should be applied and participants should be clearly instructed about the definition of a vertigo attack.

**Electronic supplementary material:**

The online version of this article (10.1007/s00415-020-10043-x) contains supplementary material, which is available to authorized users.

## Introduction

The lifetime prevalence of vertigo is reported to be 25% in the general population, with a peak prevalence in older adults up to 36% [1]. Vertigo can result from several central and peripheral vestibular disorders [2, 3]. Especially in vestibular diseases with recurrent attacks of vertigo, such as vestibular migraine, benign paroxysmal positional vertigo or Meniere’s disease, diaries can be used to get more insights into the number and nature of attacks, including symptoms, possible triggers and duration [4]. However, there are different ways to report an attack. For example, standardized questionnaires can be administered during or directly after an attack (event sampling), or attacks can be reported at the end of the day or week (time sampling). Both strategies might have their pros and cons regarding adherence (e.g., feeling too sick to report during or directly after an attack) and reliability (e.g., more recall bias when only reporting information about attacks at the end of the day). To our knowledge, it has not yet been investigated in vestibular disorders how these different strategies (event sampling and time sampling) for reporting vertigo attacks relate to each other, and whether they are comparable or complementary. This might be important since reliable reporting on number and nature of attacks can have significant implications for clinical practice and research [5].

Recently, an app-based vestibular diary was introduced, the DizzyQuest, that uses standardized questionnaires during the morning, day and evening to measure vestibular symptoms and their context. Since the DizzyQuest sample symptomatology in an individual’s flow of daily life, it has a high ecological validity [results reflect the real-life response of symptoms to real-life challenges (Martin et al. 2020, submitted)] and it does not introduce unwanted recall bias thereby increasing reliability. At least two questionnaires of the DizzyQuest specifically address vertigo attacks: the “attack questionnaire” and the “evening questionnaire”. The attack questionnaire can be used directly after an attack for standardized reporting about the number and nature of the attack. The evening questionnaire is administered at the end of the day and mainly focuses on overall symptoms during the day, including number, triggers and duration of attacks. This implies that the DizzyQuest facilitates reporting on vertigo attacks in 2 ways: directly after an attack (event sampling) and at the end of the day (time sampling). Event sampling is generally applied directly after an event, while time sampling is applied at (semi-)fixed times. Event sampling is also voluntarily initiated by the participant, while time sampling is more automatically initiated (e.g., a notification on the smartphone of the participant). Therefore, event sampling generally has a lower recall bias than time sampling, but adherence can be compromised since it is more voluntary than time sampling. Furthermore, event sampling is better at capturing very discrete attacks (with a clear beginning and an end) than less discrete attacks (e.g., some vestibular migraine attacks). These latter are better captured by time sampling [6]. Taking these pros and cons of event and time sampling into account, it could be hypothesized that both strategies might obtain different results regarding reported number, symptoms, triggers and duration of vertigo attacks.

Therefore, objective of this study was to investigate whether the number of reported vertigo attacks was equivalent between two reporting strategies: (1) event sampling using an attack questionnaire directly administered after a vertigo attack, and (2) time sampling using an evening questionnaire administered at the end of the day. Furthermore, the nature (symptoms, triggers, duration) of vertigo attacks was explored within this tested population with vestibular disorders.

## Methods

### Study population

Patients with different vestibular disorders were included at the Department of Otorhinolaryngology and Head and Neck surgery of Maastricht UMC +, HAGA Hospital, and the ‘Hoormij’ foundation, all located in The Netherlands. They were recruited from March 2019 until November 2019. Patients were included if (1) aged 18 years or above, (2) they were diagnosed with a vestibular disorder by a vestibular specialist, according to the Barany Society diagnostic criteria [7–11] (if applicable), (3) they reported having vertigo attacks, (4) they had good understanding of the Dutch language, (5) they were in possession of a smartphone or tablet running at least Android 4.0 or iOS 8.0 with an internet connection, and (6) they indicated their willingness to use the DizzyQuest as intended (e.g., complete as much questionnaires as possible). Patients were excluded if they did not feel comfortable to complete the DizzyQuest questionnaires (e.g., psychosocial questions).

### The DizzyQuest

The DizzyQuest is an app-based vestibular diary that runs from the platform “University Maastricht Experience Sampling Method (UM ESM)”, which can be downloaded for Android and iOS. This platform is an experimental version of the PsyMate™ app which facilitates the administration of multiple questionnaires and sampling schemes [12]. The DizzyQuest contains four questionnaires: a morning, day, evening and attack questionnaire (Martin et al. 2020, submitted). These questionnaires are administered daily for a certain period and examine number of vestibular symptoms, their nature (symptoms, possible triggers and duration) and psychosocial context. To assess symptom severity, the questionnaires use a seven-point Likert scale, that ranges from 1 (not at all) to 7 (very). To assess triggers and duration of vertigo attacks, multiple-choice questions are used. In this study, the attack questionnaire was voluntarily filled in directly after the occurrence of a vertigo attack (event sampling), while the evening questionnaire comprised an end-of-day vestibular diary (time sampling) that, among other topics, inquired retrospectively whether vertigo attacks were present in the past day (Online Resource 1). It should be noted that in the Dutch language, the terms “vertigo” and “dizziness” are often used interchangeably. This is in contrast with the English language, in which both terms have a separate meaning. However, when referred in Dutch to “an attack”, generally “vertigo” is meant. For consistency, this article will only use the term “vertigo”, to be consistent with the English language [3].

### Study design

Patients that fulfilled the inclusion criteria were asked by four or the authors (EM, RvdB, HB, TB) whether they wanted to participate in this study. In case of a positive response, they were contacted by two of the other authors (SvdW, AE) and instructed on how to use the DizzyQuest by means of an instructional video. All patients were instructed to fill in as many questionnaires as possible, and to always use the attack questionnaire directly after a vertigo attack. A vertigo attack was defined by each episode that the patient him- or herself would qualify as an attack of vertigo. Although large inter-individual differences exist between what patients perceive as a vertigo attack, this approach was chosen to stay as close as possible to “real-life setting”, in which patients in the future will be able to independently download the DizzyQuest on their device and might not get any instructions from a clinician regarding the definition of a vertigo attack [13].

Each evening, the evening questionnaire became available at 7:30 PM and a notification appeared on the smartphone or tablet at 8:00 PM that showed that the evening questionnaire was available to be filled in. From that moment, the evening questionnaire was available until 4 AM the next morning. If the evening questionnaire was not completed, this was considered as missing data. The attack questionnaire was always available in the DizzyQuest and could, therefore, be completed at any given moment of the day. Within this questionnaire, the patient has to indicate when the attack has stopped, to investigate the duration between occurrence of the attack and reporting (see supplementary materials).

Since inter-individual differences between included patients could exist regarding the definition of a vertigo attack, this study explicitly not investigated the appearance of vertigo attacks related to inter-individual differences (e.g., composition of baseline characteristics) or group differences (e.g., different etiologies). Main outcomes were related to differences between time and event sampling in a group of patients who had both options available for reporting. By this, they could serve as their own control.

### Patient monitoring

The ESM UM platform provides online monitoring of all activities and results of the DizzyQuest. Two authors (SvdW, AE) monitored the responses of patients. In case a patient did not start the DizzyQuest on the predetermined day, or the DizzyQuest was not completed multiple times, the patient was contacted to evaluate and to help with any problems.

### Data processing and statistical analysis

The ESM UM database was exported to Microsoft Office Excel 2007. Patients were included in the current analysis regarding vertigo attacks if they (1) reported at least one vertigo attack in the evening questionnaire, and/or (2) reported at least one vertigo attack in the attack questionnaire. The amount of days patients participated was calculated by adding up the number of days the attack and/or the evening questionnaires were filled in. The total amount of attacks reported by all patients in the evening questionnaire was determined by the sum of reported attacks during the days (answer options: 1, 2, 3 or more). In case “3 or more” was selected, the amount of attacks was registered as three, to prevent overestimation. After all, the questionnaire does not ask for the exact amount of attacks above the number of three. The answer option “3 or more” (and not the option to report any number of attacks above “3”) was chosen after careful deliberation in expert meetings and focus groups with professionals and patients (E.C. Martin et al., manuscript in preparation). In case, a patient responded in the evening questionnaire that the (first) attack of the past day, already started the day before, this was not considered as a new vertigo attack and not included in the total amount of attacks. The number of attacks reported by all patients in the attack questionnaires was determined by counting the amount of completed attack questionnaires. When comparing the amount of attacks reported at the same day in the attack and evening questionnaires, patients who filled in the attack questionnaire after the evening questionnaire were excluded. After all, the attack reported in the attack questionnaire was most likely not reported in the evening questionnaire. An exception was made for patients who completed both questionnaires within 2 min after each other. In these cases, it was hypothesized that patients most likely reported the attack in both questionnaires. To investigate the cumulative number in time of questionnaires in which at least one vertigo attack was reported, a time period of 24 days was selected. This time period was chosen since it was the average time patients participated. This facilitated investigation of a longer time period, while still having a sufficient amount of patients that could be studied. The difference in time between the cumulative number of completed evening questionnaires and cumulative number of completed attack questionnaires within this subgroup was calculated for each day by (cumulative number of attack questionnaires at day *x*—cumulative number of attack questionnaires at day *x*)/[*x*—(*x* − 1)]. Mainly descriptive statistics were applied in this study. Since triggers and duration could be reported in both the attack as evening questionnaire, Cohen’s kappa was used to analyze the level of agreement between both questionnaires regarding capturing triggers and duration of vertigo attacks [14].

### Ethical considerations

This study was in accordance with the legislation and ethical standards on human experimentation in the Netherlands and in accordance with the Declaration of Helsinki (amended version 2013). The medical ethical committee of Maastricht UMC + approved this study (2018–0809) and written informed consent was obtained from all patients, prior to participation.

## Results

### Patient characteristics and response rate

Fifty-six patients who reported having vertigo attacks participated in the study for an average of 24 days (minimum 1 day, maximum 49 days). This comprised 18 men and 36 women (missing gender *n* = 2), with a mean age of 56 years (range 26–79 years, missing age *n* = 8). Etiologies included Meniere’s disease (*n* = 24), vestibular migraine (*n* = 5), combination between vestibular migraine and Menière’s disease (*n* = 3), bilateral vestibulopathy (*n* = 9), unilateral vestibulopathy (*n* = 1), persistent postural-perceptual dizziness (PPPD) (*n* = 2), BPPV (*n* = 1), DFNA9 gene mutation (*n* = 9), and missing etiology (*n* = 2). Of these 56 patients, 29 patients used both the attack and evening questionnaires to report vertigo attacks on the same day. Eleven patients used only the evening questionnaire, eleven patients used only the attack questionnaire and five patients reported vertigo attacks in both the evening and the attack questionnaire, but on different days. All patients were included in the analysis.

According to patients’ feedback, average time to answer the evening questionnaire (the most extensive of both questionnaires) was around 2 min, after familiarization with the questionnaires.

### Reported number of vertigo attacks: attack versus evening questionnaire

**Fig. 1:**
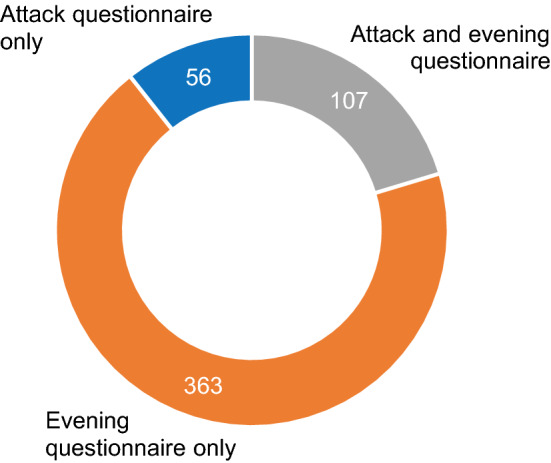
Number of at least one reported new vertigo attack in one or both questionnaires during the same day. Multiple attack questionnaires could be completed during 1 day; therefore, the total amount of attack questionnaires in this figure (*n* = 163) does not equal the total amount of completed attack questionnaires

The attack questionnaire was used 192 times to report a vertigo attack, while at least 749 new vertigo attacks were reported in 446 evening questionnaires: 206 times one attack was reported, 177 times two attacks were reported, 63 times three or more attacks were reported. Figure 1 shows that 419 times new vertigo attacks were only reported in one questionnaire during 1 day (363 evening questionnaires + 56 attack questionnaires = 419 questionnaires). It demonstrates that vertigo attacks were not always reported in both questionnaires during the same day, while both questionnaires were available to report the same vertigo attack during the same day. However, four patients reported an attack in the attack questionnaire after filling in the evening questionnaire on that particular day.

### Reporting of attacks in time: a comparison between questionnaires

**Fig. 2:**
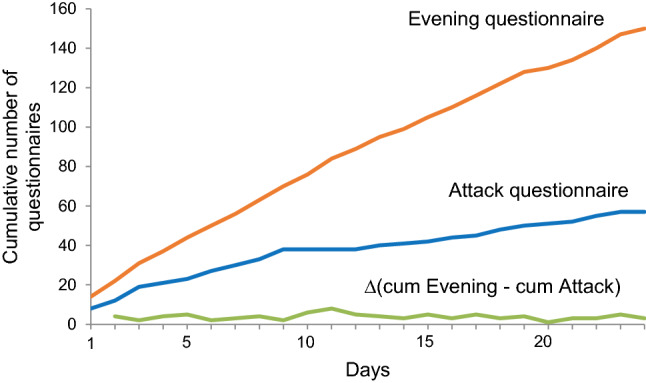
Cumulative number of questionnaires reporting at least one vertigo attack, obtained in patients who reported vertigo attacks in both the attack and the evening questionnaire, for a period of at least 24 days (*n* = 14). Δ (cum Evening—cum Attack) is the differential equation of the cumulative number of completed evening questionnaires minus the cumulative number of completed evening questionnaires in time

Figure 2 presents the cumulative number in time, of questionnaires in which at least one vertigo attack was reported. Patients were selected who reported vertigo attacks in both questionnaires for a period of at least 24 days (*n *= 14), to compare the adherence of the same patients to both questionnaires. The number of attack and evening questionnaires increases with time. The differential equation [∆(cum Evening—cum Attack)] shows that although curves of the cumulative number of both questionnaires have different slopes, the difference in reporting remained constant in time. In other words, the difference in adherence between both questionnaires remained constant in time.

### Nature of attack: results from the attack questionnaire

**Fig. 3:**
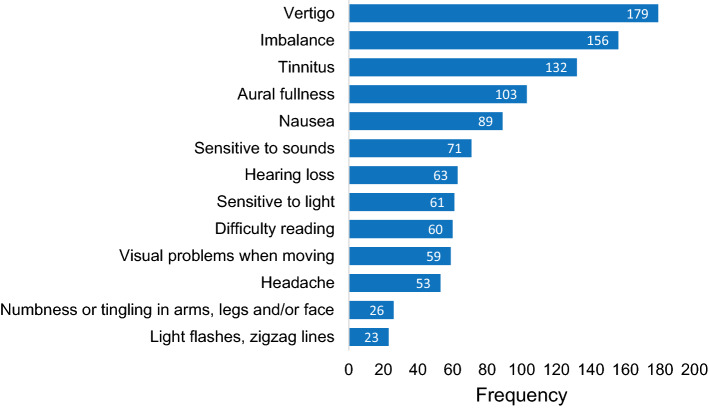
Vertigo attack symptoms, reported by 46 patients in 192 attack questionnaires. Multiple symptoms could be registered

**Fig. 4:**
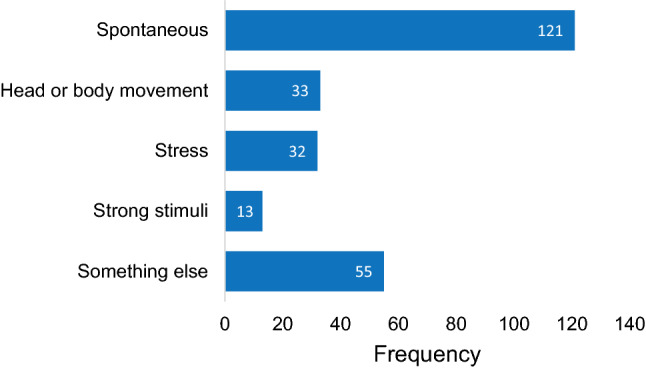
Vertigo attack triggers, reported by 46 patients in 192 attack questionnaires. Multiple triggers could be registered, including “something else” (free answer option)

**Fig. 5:**
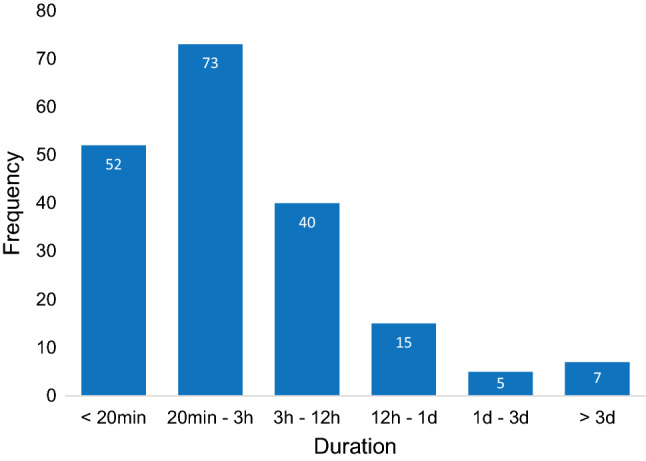
Duration of vertigo attacks, reported by 46 patients in 192 attack questionnaires

The attack questionnaire investigated the nature (symptoms, triggers, duration) of each attack. Results from the 192 attack questionnaires are presented in Figs. 3, 4 and 5. It is demonstrated that “vertigo”, “imbalance” and “tinnitus” were the most frequently reported symptoms, while visual auras like light flashes and zigzag lines were less common (Fig. 3). Regarding triggers, most attacks occurred spontaneously, but sometimes multiple triggers were identified (Fig. 4). The attack questionnaire also has the option to report an alternative trigger (“something else”), using an open question. Some alternative triggers were reported frequently, such as “lying down” (24 times). Other reported alternative triggers included, e.g., “migraine attack” (two times) and “menses” (one time). The duration varied between attacks, but most attacks lasted between 20 min and 3 h (Fig. 5). Only seven attacks lasted longer than 3 days.

### Triggers and duration compared between attack and evening questionnaires

**Fig. 6:**
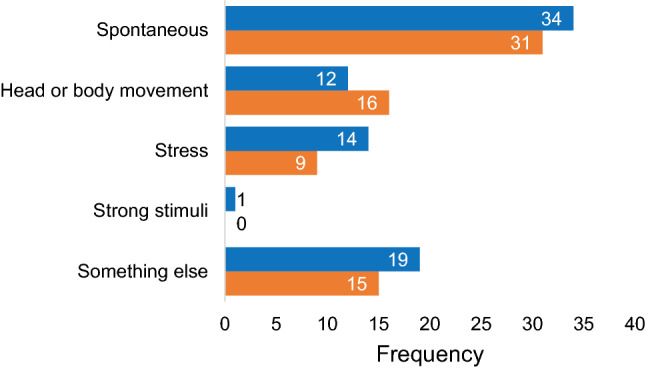
Number of vertigo attack triggers obtained in the attack questionnaire (blue) and evening questionnaire (orange) in 58 selected vertigo attacks. Multiple triggers could be registered

**Fig. 7:**
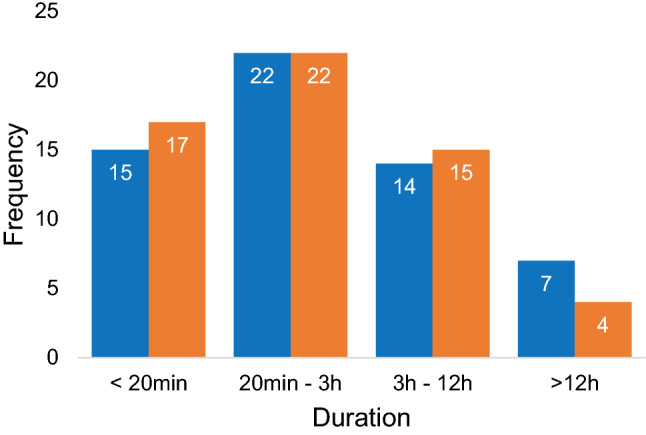
Number of vertigo attack duration obtained in the attack questionnaire (blue) and evening questionnaire (orange) in 58 selected vertigo attacks

Fifty-eight times one vertigo attack was reported in the attack questionnaire, while also one vertigo attack was reported in the evening questionnaire the same day. These vertigo attacks were selected to compare results obtained in both questionnaires since most likely both questionnaires addressed the same attack. Figures 6 and 7 illustrate the triggers and duration of these selected vertigo attacks, respectively. It shows that, although most likely the same attack was addressed in the questionnaires, reported triggers and duration were not distributed exactly the same in both questionnaires. The level of agreement between the attack and evening questionnaires on triggers was almost perfect (*κ* = 0.818, *p* < 0.001), while the level of agreement between the attack and evening questionnaires on duration was moderate (*κ* = 0.563, *p* < 0.001) [14].

## Discussion

Main objective of this study was to investigate whether the number of reported vertigo attacks was comparable between two reporting strategies: (1) event sampling using an attack questionnaire directly administered after a vertigo attack, and (2) time sampling using an evening questionnaire administered at the end of the day. It was demonstrated that within the same patient population, the number of attacks differed between these two reporting strategies: attacks were at least 3.5 times more often reported in the evening questionnaire compared to the attack questionnaire. Furthermore, triggers and duration of the same vertigo attack were sometimes described differently in both questionnaires, with a stronger agreement between both questionnaires on triggers than on duration.

Ideally, it would be expected that the number and nature of vertigo attacks would correspond almost perfectly between the attack and evening questionnaires as both questionnaires could be filled in on the same day. After all, patients were instructed to fill in the attack questionnaire directly after an attack, and to complete the evening questionnaire at the end of each day. However, only 29 out of 65 filled in both questionnaires. This study showed that the number and nature of reported vertigo attacks depend on the reporting strategy: event sampling (attack questionnaire) or time sampling (evening questionnaire). This results from the inherent differences between the two strategies that become apparent due to multiple reasons when reporting vertigo attacks: First, a vertigo attack should be discrete with a clear beginning and an end to be reliably captured by event sampling [6]. Vertigo attacks can be very discrete (e.g., some Meniere’s attacks), but other attacks might be less discrete (e.g., in vestibular migraine). In the latter cases, the concept of attack is often retrospectively reconstructed in the mind of an individual [15]. Therefore, these attacks can be missed using event sampling, and are mainly captured by time sampling. The data of the present study fit with the notion that the number of reported attacks is higher in time sampling than event sampling. Second, retrospectivity (and, therefore, recall bias) is generally lower in event sampling than in time sampling since event sampling often occurs directly after an attack, while time sampling is more likely to happen at a later stage. However, recall bias induces the tendency to report peak symptom scores rather than average symptom scores [12]. This might lead to overreporting of vertigo attack number in the evening questionnaire and might induce an increase in perceived severity of an attack. Third, event sampling is an additional workload for the participants. After all, it involves additional questions to the already present time sampling of the other questionnaires of the DizzyQuest (morning-, day- and evening questionnaires). This might be considered as a “burden” by some participants. Since they can choose whether or not to use event sampling (they have to initiate the questionnaire themselves), the experienced “burden” (e.g. feeling too sick to report during or directly after an attack) can create reactivity that results in a “learning curve” in which the reporting of vertigo attacks declines, while the attacks still appear [6]. This might lead to under-sampling of vertigo attacks. However, this phenomenon was not observed in this study (see Fig. 2). In contrast, time sampling obliges the participant to indicate whether a vertigo attack appeared or not, and also captures the non-events: periods in which it was certain that no vertigo attack appeared.

These factors show that each sampling strategy has its own pros and cons. Event sampling using an attack questionnaire probably reflects more reliably the real nature of the vertigo attack due to little recall bias. Yet, under-sampling is possible since some attacks will be missed by event sampling as they are reconstructed retrospectively, and a higher workload might induce reactivity. Time sampling using an evening questionnaire probably better captures less discrete vertigo attacks and is also able to capture the absence of vertigo attacks. Still, there is a higher risk of over-sampling and exaggeration of symptom severity. This knowledge has direct implications for clinic and research: the number and nature of reported vertigo attacks by patients depend on how their data are collected, e.g., using event sampling or time sampling.

Therefore, depending on the clinical or research question, the right strategies should be used. For example, in case little retrospectivity is desired, the attack questionnaire from the DizzyQuest might be preferred. However, if the presence and absence of vertigo attacks are considered more important, the evening questionnaire of the DizzyQuest might be preferred, especially when adherence is expected to be low in a specific population. The DizzyQuest is able to facilitate all combinations of questionnaires: attack or evening questionnaire only, or a combination of both. In clinical trails, time sampling is often preferred over event sampling due to the reasons stated above; in general, it is more reliable, less burdensome, and also captures less discrete events and non-events [6]. Nevertheless, when interpreting the obtained time sampling data, the risk of, e.g. exaggeration of symptom severity should be taken into account. It could, therefore, be proposed to combine both strategies within the same trial, in which event sampling might be used to verify the symptom severity found with time sampling. For this, both questionnaires have to be completed on the same day. However, to achieve the desired adherence to both strategies within the same trial, very strict monitoring, guidance and instruction of patients are necessary.

This study raises the question: what is the definition of a vertigo attack? After all, it was demonstrated that on an intra-individual level, differences already exist in reporting vertigo attack frequency due to different reasons, but it might be very likely that on an inter-individual level, not all patients would always consider the same cluster of symptoms of a vertigo attack [13]. Inter-individual differences between subjective symptoms and objective findings were already described using the DizzyQuest. For example, patients did not report the same amount of perceived hearing loss severity when having the same amount of hearing loss, as measured by audiometry (Martin and Verkaik et al. 2020, submitted). This study, in which patients were able to define their own “vertigo attack”, illustrates that it is imperative when using event or time sampling in clinic and research, that participants are clearly instructed beforehand on what cluster of symptoms should be reported as a vertigo attack [6]. This might seem very clear, but taking the above-mentioned considerations into account, this might not be that easy. After all, it could be hypothesized that at least three types of vertigo attacks appear: (a) very discrete vertigo attacks with a clear beginning and end (e.g., some Meniere’s attacks); (b) vertigo attacks with not a clear beginning and end, which are retrospectively reconstructed (e.g., some panic attacks); (c) symptoms reported as vertigo attacks, which are actually unfairly retrospectively reconstructed peak symptoms which were not part of an attack at all. However, future research is necessary to explore these hypotheses.

Finally, the nature of vertigo attacks as reported in this study probably reflects the selected population, including Meniere’s disease and DFNA9. It mainly shows that the DizzyQuest is able to capture a variety of symptoms.

### Limitations

Four limitations could be identified in this study. First, in the evening questionnaire, the first question concerning an attack is stated as “How many attacks of vertigo, nausea and/or hearing loss have you had today?” Although instructions were given about reporting vertigo attacks, it might still be that patients also reported attacks of only hearing loss. This might have led to a higher number of reported attacks in the evening questionnaire than in the attack questionnaire. However, based on the obtained results, this might not have explained the clear differences between both questionnaires regarding the reported number of vertigo attacks. Second, only patients with a vestibular disorder were included in this study. However, due to strict anonymization, it was not always possible to retrieve gender, age and etiology of vestibular symptoms for patients that did not completely fill in their patient details in the DizzyQuest after inclusion. Since the objective of this study did not involve research questions regarding etiology, age or gender, it was decided to still use the data of these patients. Third, this study showed a higher number of reported attacks in time sampling than in event sampling, but it could not be determined whether this was due to over-sampling or under-sampling. Fourth, this study did not investigate the difference in reported symptoms of vertigo attacks between the attack and evening questionnaires since the evening questionnaire does not facilitate reporting on the symptoms of an attack.

## Conclusion

Different methods for collecting information about vertigo attacks yield differences in reports of number and nature of attacks. Event sampling using an attack questionnaire has low recall bias and, therefore, reliably captures the nature of the attack, but induces a risk of under-sampling. Time sampling using an evening questionnaire suffers from recall bias, but seems more likely to capture less discrete vertigo attacks and it facilitates registration of the absence of vertigo attacks. Depending on the clinical or research question, the right strategy should be applied and participants must be clearly instructed about the definition of a vertigo attack.

## Electronic supplementary material

Below is the link to the electronic supplementary material.

Supplementary file1 (PDF 206 kb)

## References

[1] Gopinath B, McMahon CM, Rochtchina E, Mitchell P (2009). Dizziness and vertigo in an older population: the Blue Mountains prospective cross-sectional study. Clin Otolaryngol.

[2] Strupp M, Dieterich M, Brandt T (2013). The treatment and natural course of peripheral and central vertigo. Dtsch Arztebl Int.

[3] Bisdorff A, Von Brevern M, Lempert T, Newman-Toker DE (2009). Classification of vestibular symptoms: towards an international classification of vestibular disorders. J Vestib Res Equilib Orientat.

[4] Yeo NL, White MP, Ronan N, Whinney DJ, Curnow A, Tyrrell J (2018). Stress and unusual events exacerbate symptoms in Meniere’s disease: a longitudinal study. Otol Neurotol.

[5] Patel M, Agarwal K, Arshad Q, Hariri M, Rea P, Seemungal BM (2016). Intratympanic methylprednisolone versus gentamicin in patients with unilateral Meniere’s disease: a randomised, double-blind, comparative effectiveness trial. Lancet.

[6] Verhagen SJ, Hasmi L, Drukker M, van Os J, Delespaul PA (2016). Use of the experience sampling method in the context of clinical trials. Evid Based Mental Health.

[7] Lempert T, Olesen J, Furman J, Waterston J, Seemungal B, Carey J (2013). Vestibular migraine: diagnostic criteria: consensus document of the Barany society and the International Headache Society. Der Nervenarzt.

[8] Lopez-Escamez JA, Carey J, Chung WH, Goebel JA, Magnusson M, Mandala M (2016). [Diagnostic criteria for Meniere’s disease. Consensus document of the Barany society, the Japan Society for equilibrium research, the European academy of otology and neurotology (EAONO), the American academy of otolaryngology-head and neck surgery (AAO-HNS) and the Korean balance society]. Acta Otorrinolaringol Esp.

[9] Staab JP, Eckhardt-Henn A, Horii A, Jacob R, Strupp M, Brandt T (2017). Diagnostic criteria for persistent postural-perceptual dizziness (PPPD): consensus document of the committee for the classification of vestibular disorders of the Barany society. J Vestib Res Equilib Orientat.

[10] Strupp M, Kim JS, Murofushi T, Straumann D, Jen JC, Rosengren SM (2017). Bilateral vestibulopathy: diagnostic criteria consensus document of the classification committee of the Barany society. J Vestib Res Equilib Orientat.

[11] von Brevern M, Bertholon P, Brandt T, Fife T, Imai T, Nuti D (2015). Benign paroxysmal positional vertigo: diagnostic criteria. J Vestib Res.

[12] Mujagic Z, Leue C, Vork L, Lousberg R, Jonkers DM, Keszthelyi D (2015). The experience sampling method–a new digital tool for momentary symptom assessment in IBS: an exploratory study. Neurogastroenterol Motil Off J Eur Gastrointest Motil Soc.

[13] Newman-Toker DE, Cannon LM, Stofferahn ME, Rothman RE, Hsieh YH, Zee DS (2007). Imprecision in patient reports of dizziness symptom quality: a cross-sectional study conducted in an acute care setting. Mayo Clin Proc.

[14] Cohen J (1960). A coefficient of agreement for nominal scales. Educ Psychol Measur.

[15] Dijkman-Caes CIM, Vries MW (1991). Daily life situations and anxiety in panic disorder and agoraphobia. J Anxiety Disord.

