# Supplementary material for: The DizzyQuest: to have or not to have… a vertigo attack?
Source: J Neurol. 2020 Jul 11;267(Suppl 1):15–23. doi: 10.1007/s00415-020-10043-x (PMC7718201; doi:10.1007/s00415-020-10043-x)
Supplement: Supplementary file 1 — Supplementary file1 (PDF 206 kb) [file 415_2020_10043_MOESM1_ESM.pdf]

The DizzyQuest: To have or not to have… a vertigo attack?

Journal of Neurology

L.E.G.H. de Joode[1]*, E.C. Martin[1]*, J.J.A. Stultiens[1], C. Leue[2], P. Delespaul[2], F. Peeters[3], A. Erdkamp[4], S. van de Weijer[4], H. Blom[5], T. Bruintjes[6], A. Zwergal[7], E. Grill[8], N. Guinand[9], A. Perez-Fornos[9], M.R. van de Berg[1], J. Widdershoven[1], H. Kingma[1, 10], R. van de Berg[1, 10]

Corresponding author: R. van de Berg, raymond.vande.berg@mumc.nl

[1] Division of Balance Disorders, Department of Otorhinolaryngology and Head and Neck Surgery, Maastricht University Medical Center, Maastricht, Netherlands

[2] Department of Psychiatry and Neuropsychology, School for Mental Health and Neuroscience, Maastricht University Medical Center, Maastricht, Netherlands

[3] Department of Clinical Psychological Science, Faculty of Psychology and Neuroscience, Maastricht University

[4] mHealth, Maastricht University Medical Center, Maastricht, Netherlands

[5] Department of ENT, HagaZiekenhuis, The Hague.

[6] Apeldoorns duizeligheidscentrum, Gelre ziekenhuizen, Apeldoorn

[7] Department of Neurology, Ludwig-Maximilians-University of Munich

[8] Department of Medical Informatics, Ludwig-Maximilians-University of Munich

[9] Service of Otorhinolaryngology Head and Neck Surgery, Department of Clinical Neurosciences, Geneva University Hospitals, Geneva, Switzerland.

[10] Faculty of Physics, Tomsk State Research University, Tomsk, Russia

1.1 Evening Questionnaire

Question	Scoring system	Answer options
This was an ordinary day	Likert scale	1 = Not at all<br>4 = Moderate<br>7 = Very
I generally felt well today	Likert scale	1 = Not at all<br>4 = Moderate<br>7 = Very
I generally felt tired today	Likert scale	1 = Not at all<br>4 = Moderate<br>7 = Very
I generally felt relaxed today	Likert scale	1 = Not at all<br>4 = Moderate<br>7 = Very
I generally worried a lot today	Likert scale	1 = Not at all<br>4 = Moderate<br>7 = Very
I generally was able to concentrate well today	Likert scale	1 = Not at all<br>4 = Moderate<br>7 = Very
I felt dizzy today*	Likert scale	1 = Not at all<br>4 = Moderate<br>7 = Very
I felt nauseous today	Likert scale	1 = Not at all<br>4 = Moderate<br>7 = Very
I had balance issues today	Likert scale	1 = Not at all<br>4 = Moderate<br>7 = Very
Today I suffered from hearing loss: left	Likert scale	1 = Not at all<br>4 = Moderate<br>7 = Very
Today I suffered from hearing loss: right	Likert scale	1 = Not at all<br>4 = Moderate<br>7 = Very
Today I suffered from tinnitus: left	Likert scale	1 = Not at all<br>4 = Moderate<br>7 = Very
Today I suffered from tinnitus: right	Likert scale	1 = Not at all<br>4 = Moderate<br>7 = Very
Today I suffered from aural fullness	Likert scale	1 = Not at all<br>4 = Moderate<br>7 = Very

Today I suffered from headache	Likert scale	1 = Not at all<br>4 = Moderate<br>7 = Very
Today I suffered from light-sensitivity	Likert scale	1 = Not at all<br>4 = Moderate<br>7 = Very
Today I suffered from sound-sensitivity	Likert scale	1 = Not at all<br>4 = Moderate<br>7 = Very
Today I suffered from light flashes and/or zigzag lines	Likert scale	1 = Not at all<br>4 = Moderate<br>7 = Very
Today I suffered from numbness or tingling in arms, legs and/or face	Likert scale	1 = Not at all<br>4 = Moderate<br>7 = Very
Today I suffered from visual problems when moving	Likert scale	1 = Not at all<br>4 = Moderate<br>7 = Very
Today I suffered from difficulties reading	Likert scale	1 = Not at all<br>4 = Moderate<br>7 = Very
How many attacks of dizziness, nausea and/or hearing loss have you had today?*	Multiple choice option	- 0<br>- 1<br>- 2<br>- 3 or more
Did the first attack today already start yesterday?	Multiple choice option	- Yes<br>- No
The attacks were provoked by:	Multiple choice option<br><br>(maximum 5 options)	- Head or body movements<br>- Strong stimuli (visual, sound, busy places)<br>- Stress<br>- Nothing, started spontaneously<br>- Something else:…
If chosen 'Something else:…' What else provoked the attack?	Open answer option (maximum 120 characters)	
I was unable to function properly because of the attacks for:	Multiple choice option	1. <20 minutes<br>2. 20 minutes – 3 hours<br>3. 3 hours – 12 hours<br>4. >12 hours
To what extent were you limited in your activities today?	Likert scale	1 = Not at all<br>4 = Moderate<br>7 = Very
In which type of activities were you limited the most today?	Multiple choice option	1. Work, study<br>2. Household

	(maximum 1 option)	3. Social<br>4. Travelling, on the way<br>5. Hobbies<br>6. Sports<br>7. Sleeping

1.2 Attack Questionnaire

**Question**	**Scoring system**	**Answer options**
This attack has stopped since	Multiple choice option	1. Happening now<br>2. <20 minutes<br>3. 20 minutes – 3 hours<br>4. 3 hours -12 hours<br>5. >12 hours<br>6. Yesterday or earlier
During this attack I suffered from	Multiple choice option (maximum 13 options)	1. Dizziness*<br>2. Nausea<br>3. Imbalance<br>4. Hearing loss<br>5. Tinnitus<br>6. Aural fullness<br>7. Headache<br>8. Sensitive to light<br>9. Sensitive to sounds<br>10. Light flashes, zigzag lines<br>11. Numbness or tingling in arms, legs and-or face<br>12. Visual problems when moving<br>13. Difficulty reading
This attack was provoked by	Multiple choice option (maximum 5 options)	1. Arose automatically<br>2. Head or body movements<br>3. Strong stimuli (visual, sound, busy places)<br>4. Stress<br>5. Something else:…
If chosen 'Something else:…'<br><br>What other reason was there for the attack?	Open answer option<br><br>(Maximum 60 characters)	
How severe was this attack?	Likert scale	1 = Very mild<br><br>7 = Very severe

This attack prevented me from functioning normally for	Multiple choice option	1. <20 minutes<br>2. 20 minutes – 3 hours<br>3. 3 hours – 12 hours<br>4. 12 hours – 1 day<br>5. 1 to 3 days<br>6. >3 days

* The terms “vertigo” and “dizziness” are often used interchangeably in the Dutch language.
